# Explant cultures of *Rpe65^−/−^* mouse retina: a model to investigate cone opsin trafficking

**Published:** 2013-05-29

**Authors:** Mausumi Bandyopadhyay, Masahiro Kono, Bärbel Rohrer

**Affiliations:** 1 Department of Ophthalmology, Medical University of South Carolina, Charleston, SC; 2 Research Service 151, Ralph H Johnson VA Medical Center, Charleston, SC

## Abstract

**Purpose:**

In the absence of 11-*cis* retinal (e.g., *Rpe65^−/−^*), the chromophore for all pigments, cone opsins are mislocalized in vivo. Using the systemic application of 11-*cis* retinal, appropriate protein localization can be promoted. Here, we asked whether explant cultures of *Rpe65^−/−^* mouse retina are amenable to screening retinoids for their ability to promote opsin trafficking.

**Methods:**

Retina-retinal pigment epithelium (RPE) cultures were prepared from 7-day-old *Rpe65^−/−^ Rho^−/−^* or wild-type pups and cultured for 11 days. Explants were treated with retinoids throughout this period. Ultraviolet (UV)-opsin trafficking was analyzed by immunohistochemistry and quantitative image analysis, while its messenger RNA expression was examined by quantitative real-time PCR, and the interaction of retinoids with UV-opsin was probed in transducing-activation assays.

**Results:**

In wild-type explant cultures, UV-opsin was restricted to the outer segments, whereas in those derived from *Rpe65^−/−^ Rho^−/−^* mice, opsin trafficking was impaired. In *Rpe65^−/−^ Rho^−/−^* explants, administration of 11-*cis* retinal, 11-*cis* retinol or retinoic acid (RA) reversed the opsin trafficking phenotype. RA analogs designed to act by binding to the retinoic acid receptor or the retinoid X-receptor, however, had no effect. RA was shown to interact with the UV–cone opsin, demonstrated by its ability to effect ligand-dependent activation of transducin by UV–cone opsin. All compounds tested increased cone opsin messenger RNA expression.

**Conclusions:**

Cone-opsin trafficking defects were replicated in *Rpe65^−/−^ Rho^−/−^* retina-RPE cultures, and were reversed by 11-*cis* retinal treatment. Comparing the effects of different retinoids on their ability to promote UV-opsin trafficking to outer segments confirmed the critical role of agents that bind in the retinoid binding pocket. Retinoids that act as transcription factors, however, were ineffective. Thus, organ cultures may be a powerful low-throughput screening tool to identify novel compounds to promote cone survival.

## Introduction

Photoreceptors consist of an outer segment (OS) and inner segment (IS), cell body, and synaptic terminal. The OS contains the components of the signal transduction cascade required to turn the absorption of a photon into a change in membrane potential. In the mouse retina, cone birth peaks prenatally at embryonic day 14 (E14) [1], yet differentiation is delayed. Ultraviolet (UV)-opsin expression (documented by immunohistochemistry) starts by about postnatal day 2 (P2), whereas middle wavelength–opsin cannot be detected until P11 [2]. Early in development, the apoprotein is distributed throughout the entire cell in rods and cones, but cell membrane labeling disappears around onset of vision [2-5]. Finally, restriction of cone opsin to the OS does not occur with the initiation of growth of an OS, as cone OS formation is initiated ~P4, 10 days before eye opening [6].

Visual pigments are a covalent complex of the apoprotein opsin, a seven-transmembrane-spanning protein, and a small, light-absorbing compound, the vitamin-A-based 11-cis retinal. In mice, color vision is mediated by two cone classes containing a combination of short wavelength (UV-opsin) and/or a middle wavelength–sensitive pigment (M-opsin) [7-9], together representing 3%–5% of cells in the outer nuclear layer [10].

Rod and cone opsins are synthesized in the endoplasmic reticulum followed by posttranslational modifications occurring in both the endoplasmic reticulum and Golgi apparatus. Transport of rhodopsin involves vesicular transport from the endoplasmic reticulum through the Golgi and the formation of opsin transport carriers, followed by transport to the connecting cilium and fusion with the membrane in the IS [11]; experimental evidence suggests that cone opsin trafficking follows the same principle [12]. Rhodopsin transport to the OS and correct insertion into the disk membranes has been shown to be dependent on protein structure and folding [13]. To date, only one cone opsin folding mutation has been characterized (green opsin C203R) [14]; while this mutation leads to cone cell loss, no data on cone opsin distribution are available. Our published results [15], however, as well as those of others (e.g., [16]), suggest that 11-*cis* retinal is required for cone opsin targeting to the OS. It is of interest to note that the onset of 11-*cis* retinal production correlates with the onset of OS elongation and increased opsin production [17].

Overall, the published results suggest that 11-*cis* retinal may: 1) serve as a chaperone for all cone opsins; 2) promote proper cone-opsin folding such that chaperones can bind to the cone opsins for trafficking; and/or 3) eliminate opsin phosphorylation and arrestin-binding through action as an inverse agonist, and thereby free up the opsin for apical trafficking. Some of these concepts have been explored in reduced systems, albeit mostly using rhodopsin and its mutants. Retinoid-based chaperones have been analyzed in a cell-based system, screening for P23H rhodopsin trafficking ([18-20]; and see [21] for review); moreover, a database library of drug-like compounds has been virtually-screened in silico screening for proper fit into the binding pocket of rhodopsin, followed by P23H trafficking assay [22]. In addition, inverse agonist action has been analyzed in cell-free systems, analyzing expressed human rod and cone opsins [23,24], or isolated salamander cone photoreceptors [25]. However, experimental evidence is thus far lacking to identify the role of 11-*cis* retinal in promoting trafficking of cone opsins in intact photoreceptors.

Previously, we and others have shown that complex phenotypes can be replicated in retina culture [26-28], and that these organ cultures are amenable to pharmacological intervention [29]. In short, when early postnatal wild-type retinas are grown in culture with the retinal pigment epithelium (RPE) attached from the equivalent of P7 to P18, they recapitulate in vivo photoreceptor development, resulting in restriction of both rod and cone opsins to their respective OSs [28]. It has been confirmed that the amount of 11-*cis* retinal generated in these cultures reaches maximal levels of ~1/3 of that generated in vivo in age-matched animals, which was found to be sufficient to promote light-driven retinal activity [28]. Here, we asked whether this organ culture system could be applied to *Rpe65^−/−^* retinas to identify molecular chaperones that could promote cone survival. We first demonstrated that, as with the in vivo system, there are cone opsin trafficking defects during *Rpe65^−/−^* cone maturation, which is rescued by 11-*cis* retinal. We then screened additional retinoid-based compounds for their ability to promote cone opsin trafficking to the OSs. Our results suggest that the organ cultures may be a powerful low-throughput screening tool to not only identify novel compounds that promote cone survival, but also to analyze the mechanism of cone OS protein trafficking.

## Methods

### Animals

*Rpe65^−/−^ Rho^−/−^* mice were obtained from Mathias Seeliger (University of Tuebingen, Tuebingen, Germany) with permission from Peter Humphries (Trinity College, Dublin, Ireland) and T. Michael Redmond (National Institutes of Health), respectively. C57BL/6 mice were generated from breeding pairs obtained from Harlan Laboratories (Indianapolis, IN). Animals were housed in the Medical University of South Carolina (MUSC) Animal Care Facility under a 12h:12h light-dark cycle, with access to food and water ad libitum. All experiments were performed in accordance with the Association for Research in Vision and Ophthalmology Statement for the Use of Animals in Ophthalmic and Vision Research and were approved by the MUSC Animal Care and Use Committee.

### Retinotypic cultures

All chemicals used for organ cultures were tissue culture grade and were purchased from Invitrogen (Carlsbad, CA). Retina-RPE cultures were grown by means of the interface technique according to published protocols [26,27,30] with modifications [28]. All preparations were performed under a laminar-flow hood. Pups were deeply anesthetized by hypothermia and decapitated. Heads were rinsed in 70% ethanol and eyeballs collected and placed in ice-cold Hanks balanced salt solution plus glucose (6.5 g/l). To collect the retina with RPE, eyes were incubated in 1 ml of media containing cysteine (0.035 mg) and papain (20 units) at 37 °C for 15 min. Enzymatic activity was stopped in media plus 10% fetal calf serum. The anterior chamber was removed, followed by the lens and vitreous. Using a pair of #5 forceps, the retina with the RPE attached was then carefully dissected free from the choroid and sclera. Relaxing cuts were made into the retina-RPE sandwich to flatten the tissues. The tissues were then transferred to the upper compartment of a Costar Transwell chamber using a drop of Neurobasal medium (Invitrogen), RPE layer face down. The drop of fluid was used to flatten out the retina by gently spreading the drop of liquid with the fused end of a glass Pasteur pipette. Neurobasal media with 1% N1 and 2% B-27 supplements was placed in the lower compartment. The cultures were kept in an incubator (5% CO_2_, balanced air, 100% humidity, at 37 °C). The medium was changed every two days. No antimitotics or antibiotics were required. Additives (e.g., retinoids) were added to the media in the lower compartment and were replaced at each medium change. Handling of retinoids was performed under dim red light illumination to avoid bleaching.

### Compounds

Additives were replaced with each media change. 11-*cis* and all-*trans* retinal were generously provided by Rosalie Crouch (Medical University of South Carolina, Charleston, SC). A dose-response curve comparing the protective effects of 11-*cis* retinal (i.e., increasing trafficking of cone opsins to the cone OSs) versus the cytotoxic effects over a range of 1 nM to 10 μM revealed 1 μM as the preferred dose (data not shown). Based on the 11-*cis* retinal results, 11-*cis* retinol was also tested at 1 μM. Likewise, all-*trans* retinoic acid (RA) at high concentrations is toxic [31], but a dose-response curve revealed that the 500 nmol concentration in culture conditions promotes cone opsin trafficking (data not shown). The two retinoid receptor agonists, RARα-selective Am80 [4-(5,6,7,8-tetrahydro-5,5,8,8-tetramethyl-2-naphthylaminocarbonyl)benzoic acid] and RXR-selective SR11345 [4-[1-(5,6,7,8-tetrahydro-3,5,5,8,8-pentamethyl-2-naphthalenyl)-2-methylpropenyl]benzoic acid] were generously provided by Marcia Dawson (Burnham Institute for Medical Research, La Jolla, CA), and both were used at 1 µM as recommended [32]. We will refer to them as RAR- and RXR-agonists, respectively, throughout the manuscript. The level of endogenous retinoids, the retention rate of the exogenous compounds, and the stability of the different exogenous compounds in the supernatant and the organ cultures—all of which could potentially affect the experimental readouts—were not determined. These potential effects are very difficult to address experimentally, and are beyond the scope of the manuscript.

### Immunohistochemistry

Retina cultures were fixed in 4% paraformaldehyde. For sections, tissues were cryoprotected in 30% sucrose, frozen in TissueTek O.C.T. (Fisher Scientific, Waltham, MA) and cut into 14 μm cryostat sections [26,33,34]. After the slides were washed in phosphate buffered saline (PBS; in mM: 2.7 KCl, 138 NaCl, 6.6 Na_2_HPO_4_(H_2_O), 1.8 KH_2_PO_4_) they were blocked with 10% normal goat serum and 3% bovine serum albumin (in PBS containing 0.4% triton X). Tissues were incubated overnight in blocking solution containing the UV-opsin antibody (generously provided by Jeannie Chen, University of Southern California, Los Angeles, CA), followed by incubation with the appropriate fluorescently-labeled secondary antibody for 4 h (Molecular Probes, Carlsbad, CA). Control experiments included omission of primary antibody and observation of singly labeled slides through the appropriate filter set. Sections were mounted and analyzed by confocal microscopy (Leica, Bannockburn, IL) using identical settings for all slides. Images were imported into Adobe^®^ Photoshop^®^ software (San Jose, CA) for further analysis. To quantify antibody distribution, images were thresholded and distribution profiles analyzed using Image-J software, as previously reported [15]. The mean values of the antibody distribution profiles were plotted in Origin^®^ software (Northampton, MA), and individual profiles were compared using repeated measure analysis of variance and a post hoc Fisher test using Statview^®^ v. 5.0 software (SAS Institute; Cary, NC). For immunohistochemical analysis, we examined 20–30 cones per section, averaging 3–6 organ cultures per compound.

### Quantitative real-time polymerase chain reaction

Equal amounts of RNA (2 µg, derived from four pooled cultures) were used in reverse-transcription reactions (Invitrogen) as described previously [35]. In short, RNA (2 μg each) were used to generate cDNA in reverse transcription reactions (Invitrogen). PCR amplifications were conducted using the QuantiTect Syber Green PCR Kit (Qiagen) with 0.2 µmol/l forward and reverse primers and equal amounts of complimentary DNA using primers for β-actin (forward 5’-GCT ACA GCT TCA CCA CCA CA-3’, reverse 5’-TCT CCA GGG AGG AAG AGG AT-3’) and UV-opsin (forward 5’-TTG GGC TCT GTA GCA GGT CT-3’, reverse 5’-CAA GTA GCC AGG ACC ACC AT-3’). Reactions were treated with 0.01 U/µl AmpErase^®^ UNG enzyme (Applied Biosystems) to prevent carryover contamination. Real-time PCR were performed in triplicate in a GeneAmp^®^ 5700 Sequence Detection System (Applied Biosystems) with the following cycling conditions: 50 °C for 2 min, 94 °C for 15 min, 40 cycles of 94 °C for 15 s, and 58 °C for 1 min. Quantitative values were obtained from the cycle number (C_t_ value), establishing the fold difference in gene expression between the treated and untreated retina cultures. Each compound was tested 3–5 times.

### Transducin activation assay

To determine whether RA is able to directly interact with mouse UV cone opsin, we assessed the ability of the opsin to activate transducin with and without RA. Mouse UV cone opsin gene (Origene Technologies, Rockville, MD) was extended with the codons for the last eight residues of the bovine rhodopsin C-terminus, the 1D4 epitope [36], and inserted into the modified pMT-2 expression vector [37] as an EcoRI-NotI cassette. This gene was transiently transfected into green monkey kidney cells (COS-1 cells) essentially using the method described by Oprian [38]. On the third day after transfection, COS-1 cells were harvested and membranes prepared using a discontinuous sucrose gradient as described previously [39,40]. Transducin was purified from bovine retinas [41,42]. The ligand-dependent ability of mouse UV-opsin to activate transducin was measured using a radioactive filter-binding assay in the presence and absence of RA (20 or 200 µM). Opsin (~1–10 nM) and transducin (2.5 µM) were mixed with the reaction buffer (10 mM 2-(N-morpholino)ethanesulfonic acid, 100 mM NaCl, 50 mM MgCl_2_, 1 mM dithiothreitol (DTT), pH 6.4) and ligand (0, 20, or 200 µM) added 1 min before addition of guanosine 5’-O-[gamma-thio]triphosphate (GTPγS; 3 µM containing 1 µCi GTPγS^35^) in a total volume of 100 µl as described by Kono and Crouch [23]. At 1 min intervals, 10 µl aliquots were transferred to nitrocellulose filter membranes on a vacuum manifold. Filter membranes were then transferred to vials with 10 ml beam scintillation counter (BCS) liquid scintillation fluid (Amersham; now GE Healthcare Biosciences, Piscataway, NJ). The assay was conducted under dim red light conditions. Radioactivity was converted to moles by counting aliquots of 30 pmol GTPγS and used as the conversion factor for the assay. Assuming pseudo-first order kinetics, we determined activity from the slope of the time course of the reaction. Basal transducin activity at this pH was determined in the absence of opsin by using membrane preparations of mock-transfected COS-1 cells. Activity reported here has this basal activity subtracted.

### Statistics

Data are presented as mean ± standard error of the mean (SEM). For data consisting of multiple groups, one-way analysis of variance followed by Fisher’s post hoc test (p<0.05) was used; single comparisons were analyzed by *t* test analysis (p<0.05), and quantitative real time (RT)–PCR data was compared by *z* test analysis (p<0.05).

## Results and Discussion

### Cone phenotype in retinal pigment epithelium-specific protein 65kDa and rhodopsin (*Rpe65^−/−^ Rho^−/−^*) mice is recapitulated in organ culture

**Figure 1:**
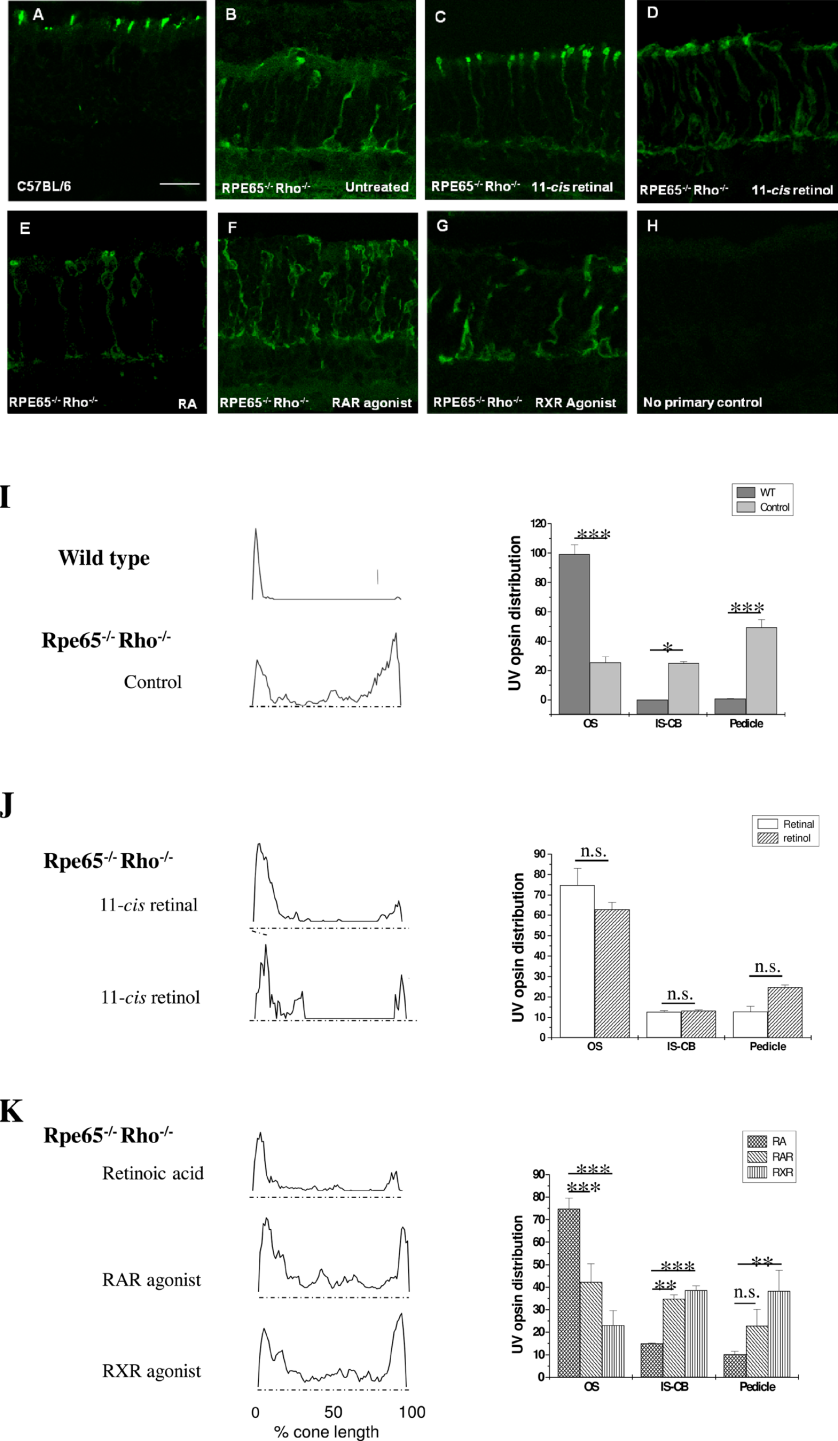
Ultraviolet–cone opsin localization in wild-type and *Rpe65^−/−^ Rho^−/−^* explant cultures. **A**: In wild-type cultures, the ultraviolet (UV)-opsin proteins are trafficked to the photoreceptor outer segment (OS), whereas (**B**) in retinal pigment epithelium-specific protein 65kDa and rhodopsin (*Rpe65^−/−^ Rho^−/−^*) explant cultures treated with vehicle, UV-opsin is found throughout the cone photoreceptor, mostly in the axon and synapse, with a minor amount trafficked correctly to the cone OS. **C**: Exogenous supplement of 1 μmol 11-*cis* retinal, or (**D**) 1 μmol 11-*cis* retinol to *Rpe65^−/−^ Rho^−/−^* explant cultures improved trafficking of UV–cone opsin toward the target site. Trafficking of UV–cone opsin in *Rpe65^−/−^ Rho^−/−^* explant cultures did not improve significantly upon treatment with (**E**) retinoic acid (RA; 500 nmol), (**F**) retinoic acid receptor (RAR)-agonist (AM80, 1 μmol) or (**G**) retinoid X-receptor (RXR)-agonist (SR11345, 1 μmol). **H**: No primary antibody control is provided to document non-specific binding of the secondary antibody. **I**: UV–cone opsin distribution profiles and quantitative assessment in wild-type and *Rpe65^−/−^ Rho^−/−^* explants. Protein distribution profiles were obtained from binarized and thresholded images normalized to a fixed size using Image J software. The protein intensity distribution was analyzed based on three different cone compartments: OS; inner segment (IS), cell body and axon; and pedicle. The mean distribution profile is plotted for the UV–cone opsin, for which examples were shown in (**A**-**H**). The data confirmed that UV–cone opsin is trafficked exclusively to the OS in the wild-type explant. In *Rpe65^−/−^ Rho^−/−^* explants, ~25% of the cone opsin is localized to the OS. **J**: Cone opsin trafficking was increased to ~75% after 11 *cis*-retinal and ~65% after 11-*cis* retinol treatment. Image acquisition and analysis was performed as in (**I**). **K**: Cone opsin trafficking to the outer segments was increased to ~75% after RA treatment, whereas treatment with RAR- or RXR-agonist had no effect. Please note that none of the comparisons between RAR and RXR were statistically significant and are not identified as such. Image acquisition and analysis was performed as in (**I**; n=12–18 per condition; *p<0.05; **p<0.01; ***p<0.005; p>0.05 or n.s. not significant). Scale bar=20 μm.

In earlier studies [15,43,44], we described the requirement of 11-*cis* retinal for localization of cone opsin and cone OS membrane proteins to the cone OS of the mouse retina. Such localizations of cone OS proteins might confer integrity to the OS and would promote cone-based vision. In retina-RPE-explant cultures derived from wild-type mice, sufficient 11-*cis* retinal is generated to promote both proper cone OS protein (UV–cone opsin and cone transducin) trafficking as well as light-driven retinal activity [28]. Here, we tested whether explants from *Rpe65^−/−^ Rho^−/−^* mice grown in culture from the equivalent of P7 to P18 recapitulate the UV–cone opsin trafficking defect observed in vivo*.* The *Rpe65^−/−^ Rho^−/−^* mouse retina was used to eliminate rhodopsin as a sink for retinoids, an approach we have successfully used in vivo to show that ligand is required during cone opsin synthesis for successful trafficking [15]. Distribution profiles for UV-opsin as identified by immunohistochemistry (Figure 1A,B) were plotted (Figure 1I, left-hand panel) and quantified using densitometry (Figure 1I, right-hand panel) [15]. While UV-opsin is restricted to the cone OSs in explants from wild-type mice (Figure 1A,I; see also reference [28]), it was distributed throughout the entire cone cell with only ~25%–30% being properly localized to the OSs in *Rpe65^−/−^ Rho^−/−^* explants as previously shown in vivo (Figure 1B,I) [15,44]. Thus, in this in vitro system, we can now analyze retinoid-based compounds for their ability to promote UV-opsin protein trafficking.

### 11*-cis* retinal corrects ultraviolet-opsin localization in *Rpe65^−/−^ Rho^−/−^* retina explants

**Figure 2:**
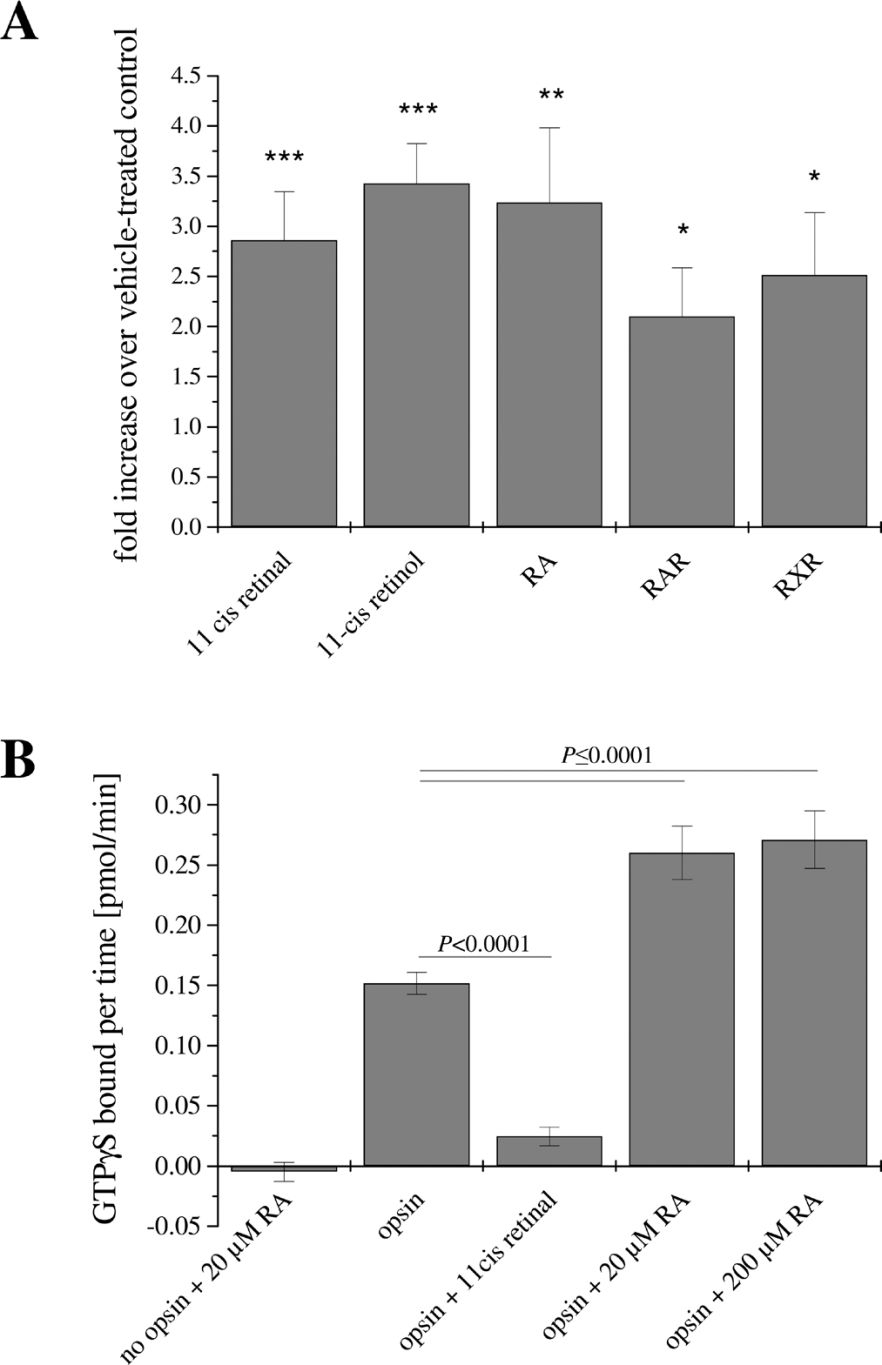
Analysis of retinoic acid as a transcription factor or retinoid analog. **A**: Relative expression of ultraviolet (UV)-cone opsin messenger RNA (mRNA) in retinal pigment epithelium-specific protein 65kDa and rhodopsin (*Rpe65^−/−^ Rho^−/−^*) explants. Explants were treated with 11-*cis* retinal, 11-*cis* retinol, retinoic acid (RA), retinoic acid receptor (RAR)-agonist and retinoid X-receptor (RXR)-agonist, respectively. An equal amount of messenger RNA (mRNA) was used from each group for quantitative RT-PCR and normalized to β-actin. All compounds increased UV–cone opsin mRNA expression (*p<0.01; **p<0.001; ***p<0.0001). **B**: Transducin activation by the mouse UV cone opsin in the absence and presence of 11-*cis* retinal and all-*trans* retinoic acid (RA) was examined. Activity has been corrected for intrinsic transducin activity in the absence of opsin and ligand using mock-transfected green monkey kidney cell (COS-1) membranes. RA was also added to the mock-transfected COS-1 cell membranes to ensure any ligand had no effect on endogenous COS-1 cell proteins. The RA concentrations used were 20 and 200 µM. As expected, 11-*cis* retinal was found to act as an inverse agonist for mouse UV–cone opsin; RA was found to act as an agonist for UV–cone opsin, increasing transducin activity using either one of the two concentrations.

*Rpe65^−/−^ Rho^−/−^* mouse explant cultures were first treated with 11-*cis* retinal (1 µM) to examine whether UV-opsin localization to the OS could be improved (Figure 1C). Incubating *Rpe65^−/−^ Rho^−/−^* cultures with 11-*cis* retinal resulted in an almost wild-type distribution profile for UV-opsin with ~75% of cone opsin restricted to the OS (Figure 1C,J). UV-opsin messenger RNA (mRNA) was also increased in the presence of 11-*cis* retinal when compared to untreated controls (Figure 2A), as previously shown in the intact *Rpe65^−/−^ Rho^−/−^* mouse [15], presumably due to the increased need for protein in the presence of stabilized OSs. Our recent experiments, analyzing cone OS development in the *Nrl^−/−^ Rpe65^−/−^* retina have confirmed using electron microscopy that 11-*cis* retinal treatment leads to the formation and stabilization of the cone OS [45].

### 11*-cis* retinol can be used for ultraviolet-opsin trafficking in *Rpe65^−/−^ Rho^−/−^* retina explants

11-*cis* retinal is synthesized in the eye by two mechanisms; the classical visual cycle of the RPE, which utilizes all-*trans* retinol as a substrate to convert it into 11-*cis* retinal in an RPE-65-dependent fashion, and the alternative visual cycle comprised of the Müller and cone photoreceptor cells, in which the bleached all-*trans* retinol is isomerized into 11-*cis* retinol by the Müller cells and subsequently oxidized in the cones to 11-*cis* retinal [46]. Here, we asked whether the juvenile *Rpe65^−/−^* cones can utilize 11-*cis* retinol to promote UV-opsin trafficking. The addition of 11-*cis* retinol (1 µM) resulted in a distribution profile with ~65% of cone opsin restricted to the OS (Figure 1D,J). Cone opsin trafficking to the OS was indistinguishable between 11-*cis* retinal and 11-*cis* retinol (p=0.2). Ala-Laurila and colleagues [25] have shown that 11-*cis* retinol does not serve as an inverse agonist for blue cone opsins, and that in all cone cells, retinol treatment yielded pigment, indicating that in cones, 11-*cis* retinol is converted to 11-*cis* retinal. Therefore, since cultured UV cones can utilize 11-*cis* retinol, this suggests that the appropriate 11-*cis* retinol dehydrogenase (not yet identified) is present and operative. As for the 11-*cis* retinal experiments, UV-opsin mRNA was also increased in the presence of 11-*cis* retinol when compared to untreated controls (Figure 2A).

### All-*trans* retinoic acid improves ultraviolet-cone opsin localization in *Rpe65^−/−^ Rho^−/−^* retina explants

Another retinoid whose levels change during retinal development [47], is influenced by light exposure [48], and could serve as a source to generate *cis*-retinoids [49] is RA (see discussion below). Treatment of the *Rpe65^−/−^ Rho^−/−^* retinal explants with 500 nM RA resulted in significantly improved UV–cone opsin (~75% is properly localized in OS) trafficking (Figure 1E,K); these levels were indistinguishable from those generated by 11-*cis* retinal or 11-*cis* retinol (Figure 1J).

The main role of RA is that of a transcription factor, acting via two different RA-receptors, the retinoic acid receptor (RAR) and the retinoid X-receptor (RXR; now also referred to as the rexinoid receptor; reviewed by [50]). To examine the possibility that the RA-mediated effects on UV–cone opsin trafficking might have been mediated by an increase in expression of a chaperone or trafficking protein, organ cultures were treated with 1 µM RAR- or RXR-agonist. Immunohistochemical analyses of retina explants exposed to either the RAR- or RXR-agonist and stained for UV-opsin showed that the RA analogs had no effect on cone-opsin localization (Figure 1F,G). The cone opsin distribution profile in cone photoreceptors (Figure 1K) revealed that only ~40% and 20% of the UV-opsin protein was localized to the OS, respectively, in RAR- and RXR-agonist-treated explants. The remainder of the protein was distributed throughout the IS, cell body, axon, and pedicle. Quantitative RT–PCR confirmed, however, that the agonists of RAR and RXR, as well as RA, significantly increased UV–cone opsin expression in retina explants by approximately 2.5-fold (Figure 2A). UV-opsin expression is known to be induced by RA. Taken together, transcription factor–mediated processes do not appear to be responsible for the RA-mediated effects on UV–cone opsin trafficking.

Alternatively, the trafficking data suggest that RA might interact with the chromophore-binding pocket of UV–cone opsin. To examine this hypothesis, we examined the effects of RA on the ability of mouse UV–cone opsin to activate its G-protein, transducin, using a radioactive filter-binding assay [23]. As shown previously for red cone opsin [23], 11-*cis* retinal (1 µM) acts as an inverse agonist, deactivating the mouse UV–cone opsin (p<0.0001). RA, on the other hand, did increase mouse UV-opsin activity significantly (20 µM, p=0.0001; or 200 µM, p<0.0001), suggesting that RA can interact directly with the UV-opsin protein by acting as an agonist (Figure 2B). It is of interest to note that, using the same transducin assay, Kono and Crouch have shown that retinoids with polyene chains extended in a *trans* configuration beyond the 9-carbon position act as agonists for the red cone opsin [24]. Since RA was found to be able to bind to isolated recombinant UV–cone opsin, the RA effect on cone opsin localization in the organ cultures appears to be due to RA associating with the retinoid-binding pocket, and thereby aiding in protein folding and transport. However, in organ cultures, RA was in an environment where its stability could not be guaranteed, and it is possible that RA itself was not responsible for the beneficial effects seen in organ cultures. Due to the limited scope of this manuscript, this was not explored further experimentally.

Urbach and Rando have shown that all-*trans* RA can be converted on liver membranes into 9-*cis* RA in a nonenzymatic reaction mediated by thiol groups [49], which could then easily be reduced to 9-*cis* retinol followed by a second dehydrogenase reaction to 9-*cis* retinal, the latter of which is an effective ligand for all opsins. Whether this thiol-dependent conversion of all-*trans* into 9-*cis* RA is a ubiquitous mechanism present in all tissues remains to be examined. Alternatively, RA or its conversion products might bind to a secondary binding site aiding in folding and transport. Secondary binding site(s) for all-*trans* retinal have previously been inferred, and are thought to be based on Schiff bases to lysine residues other than Lys^296^ and/or to lipids (see [51] for review), although the effects that this kind of binding might have upon protein folding have not yet been examined.

Interestingly, Weiler and Vaney have uncovered transcription-factor-independent effects of RA in the retina. RA appears to be involved in modulating gap-junction permeability between horizontal cells of the mammalian retina, and RA production increases with the level of illumination [48]. While it is unclear how RA is generated in the retina, it has been suggested that RA is generated in the postnatal mouse retina by enzymes that convert retinaldehyde to RA, since after P10, when significant amounts of 11-*cis* retinal were present in the retina, light stimulation increased RA synthesis significantly in an age-dependent manner over the dark-adapted level [52]. The process may be similar to that found in rat liver microsomes and cytosol, which have been shown to convert 11-*cis* retinol and 11-*cis* retinal into RA [53]. Finally, since the affinity for the endogenous ligand 11-*cis* retinal is significantly higher than that for RA, this interaction may not occur in vivo. Nevertheless, the data may prove useful in the ongoing examination of the chromophore-binding region of the cone opsins [24].

### Conclusions

In summary, we have established that cone development progresses normally in wild-type retina-RPE explant cultures; however, cone OS proteins do not become restricted to the OS by P18 in *Rpe65^−/−^ Rho^−/−^* explants. As in the whole animals, the addition of 11-*cis* retinal promoted cone OS maturation. Thus, the organ culture system might be a powerful tool to identify compounds for their ability to help traffic cone opsin to the OSs, thereby stabilizing and protecting cones. For proof of concept, we examined 11-*cis* retinol, all-*trans* RA, and its receptor analogs for their ability to promote cone OS maturation. In this process, we indirectly confirmed that the alternative retinoid pathway is operational already in the juvenile retina; we uncovered that all-*trans* RA, acting as an opsin agonist, can promote UV–cone opsin trafficking; finally, we suggest that RA-mediated transcription, while leading to increased UV–cone opsin and transducin expression, does not increase chaperones that can mitigate cone OS protein mislocalization and accumulation.
